# Factors influencing cultivated ginseng (*Panax ginseng* C. A. Meyer) bioactive compounds

**DOI:** 10.1371/journal.pone.0223763

**Published:** 2019-10-16

**Authors:** Han Yu, Jiaxin Zhao, Jian You, Jiangnan Li, Hongyu Ma, Xia Chen

**Affiliations:** 1 College of Agriculture, Jilin Agricultural University, Changchun, Jilin, China; 2 National & Local United Engineering Laboratory for Chinese Herbal Medicine Breeding and Cultivation, Jilin University, Changchun, Jilin, China; 3 School of Life Sciences, Jilin University, Changchun, Jilin, China; 4 Jilin Provincial Joint Key Laboratory of Changbai Mountain Biocoenosis and Biodiversity, Academy of Science of Changbai Mountain, Yanbian, Jilin, China; Higher Institute of Applied Sciences and Technology of Gabes University of Gabes, TUNISIA

## Abstract

We aimed to investigate the effects of genome, age, and soil factors on cultivated *Panax ginseng* C. A. Meyer (CPG) compounds under identical climate and agronomic practices. Eight populations of CPG from different years and rhizosphere soils were collected from garden and cropland in the city of Ji’an, China. Inter-simple sequence repeat (ISSR) primers were used to detect genetic diversity and identity, and soil microbial community diversity. Soil enzyme activities and nutrients were also measured. The contents of total ginsenosides (TG), Rg1, Re, Rf, Rd, and ginsenoside extractions of CPG were analyzed by spectrophotometry and HPLC. The relative importance of each factor was analyzed by mathematical methods such as correlation analysis, stepwise line regression, and path analysis. Regression equations of similarity values of HPLC fingerprint (SVHF), richness index of HPLC fingerprint (RIHF) and the TG, Rg1, Re, Rf, and Rd contents with their respective significant correlation factors were obtained. For SVHF, the relative importance is age>microbial community diversity>genetic diversity. For RIHF, the relative importance is age>genetic diversity>microbial community diversity. For TG, Rg1, and Rf contents, the relative importance is age>microbial community diversity. Ginseng age and genetic identity influenced Rd content, and age was more important. Total phosphorus was the only directly negative effect on Re. According to regression equations and path analysis, increasing age and decreasing Shannon (H') could improve the TG, Rg1, and Rf contents, with little effect on SVHF. Adding age, genetic diversity, and decreasing Shannon (H’) increased RIHF. Adding age and genetic identity could also improve Rd content. Appropriate decreases in total phosphorus might increase Re content. These findings are significant for CPG scientific cultivation methods, through which CPG bioactive ingredients could be finely controlled via regulation of genotypes and cultural conditions.

## Introduction

For at least 2,000 years, *Panax ginseng* C. A. Meyer, a perennial herb in the *Araliaceae* family commonly known as Asian ginseng [1], has been valued as an herbal tonic and stimulant in China [2,3]. *P*. *ginseng* is widely cultivated in northeast China, Japan, Russia, and the Korean peninsula [4]. There are two main cultivated types, garden ginseng (GGS) and cropland ginseng (CGS). GGS is grown by traditional cultivation methods by sowing *P*. *ginseng* seeds into a garden after deforestation and reclamation. Under purely artificial conditions, their growth usually spans 4–7 years. CGS includes only sowing seeds of *P*. *ginseng* into cropland, and its cultivation techniques are the same as GGS. Ji’an is located in southeast Jilin Province, China, and its climate data are shown in S1 Table. The region’s climate and soil permeability are suitable for the growth and development of ginseng.

The major bioactive ingredients of *P*. *ginseng* are a group of triterpene saponins known as ginsenosides [5,6,7]. More than 30 ginsenosides have been isolated from ginseng roots and are classified into two main groups, the glycosides of 20(S)-protopanaxadiol (Rb1, Rb2, Rc, Rd, Rg3, and Rh2) and the glycosides of 20(S)-protopanaxatriol (Re, Rf, Rg1, Rg2, Rh1, and R1) [8,9]. Ginsenosides have extensive pharmacological action including neuroprotective [10,11,12], anti-aging [13,14], immunomodulatory [15,16], cardiovascular protective [17,18], anti-tumor [19,20,21], and internal secretion adjustment effects [22,23]. Different ginsenosides can have completely different biological activities and pharmacological effects [24,25,26]. For example, Rg1 has a role in angiogenesis, while Rb1 inhibits the earliest step of angiogenesis [27]. The composition and content of ginsenosides is the most important factor affecting the ginseng medicinal value. However, TG content in different ginseng roots can vary by up to 20% [28]. Assessments of published literature reveals a poor understanding of the factors influencing composition and content of ginsenosides in ginseng roots, including age, genotype, soil factors, cultivation methods, and preservation or extraction methods [8,29]. If we understand the relative contribution of genotype, age, and soil factors to the variations in cultivated *Panax ginseng* C. A. Meyer (CPG) total ginsenosides (TG) composition, then scientific cultivation methods could be established.

The objective of our study was to find a quantitative relationship between genome, ginseng age, soil factors, and the composition and content of ginsenosides in CPG roots under the same climate and agronomic practices. We focused on Ji’an, where CPG is reputed to be produced at high quality and sold at premium prices. In addition, the experimental results will contribute to establishing best scientific cultivation methods to improve and control the quality and yield of ginseng roots.

## Materials and methods

### Plant materials

A total of 126 plants, which corresponded to eight cultivated populations of *P*. *ginseng* (CGS: 4, GGS: 4), were taken from Taishang in Ji’an, Jilin province, China in 07/2011 (Table 1). We confirmed that permits were obtained from Yisheng Pharmaceutical Company where collecting took place. We also confirmed that the location accessed was not privately owned and the field studies did not involve endangered or protected species. Fresh leaves were collected, dried in plastic bags with silica gel, transported back to the laboratory, and kept at −80°C. At the same time, the soil adhered to the surface of the roots (rhizosphere soil) was collected and put in sterile polyethylene bags, transported back to laboratory, and kept at −20°C. Within one day of root collection, roots were rinsed with tap water to remove soil, blotted dry, and then dried in plastic bags with silica gel. After drying, the whole roots (containing secondary roots and storage roots) of each population were prepared for analysis by grinding to a fine powder with a tissue grinder (KX-11A/B/C, Ji’nan Kexiang Instrument Co., Ltd., China). Powdered samples were stored at room temperature in plastic bags.

**Table 1:** Cultivated type, age, and sample sizes of cultivated *P*. *ginseng* populations.

Populations	Ginseng age (year)	Longitude/Latitude	Height above sea level (m)	Sample size
CGS I	1	41°10'13.2''N/125°55'13.0''E	503	16
CGS II	2	41°09'63.6''N/125°55'48.8''E	503	15
CGS III	3	41°10'14.4''N/125°05'35.2''E	503	16
CGS IV	4	41°10'17.6''N/125°55'37.5''E	511	15
GGS I	1	41°12'19.7''N/125°58'19.9''E	630	16
GGS II	2	41°12'23.5''N/125°58'30.5''E	655	16
GGS III	3	41°12'13.7''N/125°58'09.9''E	643	16
GGS IV	4	41°12'18.1''N/125°58'21.5''E	678	16

### DNA extraction

Total genomic DNA was extracted from leaves by using Plant Genomic DNA Isolation Kit (NEP003-1, Beijing Dingguo Changsheng Biotechnology Co., Ltd., China). DNA concentration was then determined by comparing the plant DNA samples with commercial standard lambda DNA on 0.8% (w/v) agarose gel, after which it was adjusted to 5 ng/μl.

### ISSR-PCR amplification

ISSR primers used in this study were synthesized by Beijing Dingguo Changsheng Biotechnology Co., Ltd (China), according to the primer set published by the University of British Columbia (UBC). One hundred ISSR primers were initially screened, and twelve that yielded bright and discernible bands, were used for the analysis of all 126 samples (Table 1). Fifteen or sixteen individuals from each population were used for the primer screening, and PCR amplifications were repeated for working primers to check the stability and reproducibility of ISSR fragments. PCR was performed in 25 μl reactions containing 1.75 mM MgCl_2_, 0.25 mM dNTPs, 1 U Taq DNA polymerase (TaKaRa), 0.2 μM primers and 10 ng DNA templates. PCR amplifications were performed in the Mastercycler Gradient PCR (Eppendorf, Germany) with the following program: initial denaturation at 94°C for 5 min; 40 cycles of 94°C for 50 s, appropriate annealing temperature (see Table 2) for 45 s, 72°C for 1 min; and final synthesis at 72°C for 10 min. A negative control with no DNA added was included in each PCR run. Amplification products were separated with 1.5% agarose gels (1×TAE buffer) at 80 V for 1.5 h, stained with ethidium bromide (0.5 μg/ml), and photographed under UV light using an EC3 Gel Documentation System (UVP, USA).

**Table 2:** Polymorphisms of inter-simple sequence repeat markers in cultivated *P*. *ginseng* populations.

Primer code	Sequence^a^	*T*_*A*_ (°C)^b^	Size range (Kb)	*N*_*PL*_*/N*_*L*_^c^	Ppl (%)^d^
UBC807	(AG)8T	55.4	210–2,200	26/26	100%
UBC808	(AG)8C	58.5	200–2,000	19/16	84.20%
UBC809	(AG)8G	60.2	200–2,070	23/18	78.30%
UBC815	(CT)8G	50.7	310–1,510	13/10	76.90%
UBC823	(TC)8C	53.9	310–1,820	19/18	94.70%
UBC826	(AC)8C	61.7	300–1,870	23/23	100%
UBC834	(AG)8YT	53.9	180–2,030	22/22	100%
UBC836	(AG)8YA	55.4	180–1,110	14/13	92.90%
UBC840	(GA)8YT	55.4	190–2,000	20/18	90%
UBC856	(AC)8YA	52.2	230–1,500	18/16	88.90%
UBC866	(CTC)6	62.9	320–2,100	18/18	100%
UBC868	(GAA)6	50.2	210–1,890	17/14	82.40%

^a^Y = C/T

^b^*T*_*A*_: Annealing temperature (°C)

^c^*N*_*L*_: Number of loci scored, *N*_*PL*_: Number of polymorphic loci scored

^d^Ppl: Percentage of polymorphic loci

### Extraction of ginsenosides

The extraction method of ginsenosides as based on the protocol by Lim W et al., with some modifications [30]. An accurately weighed sample (100 mg) of each population’s roots was transferred to a 50 ml centrifuge tube. Ginsenosides were extracted in 20 ml of 100% HPLC-grade methanol and placed in a sonicator bath for 15 min at 60°C. The sample tube was centrifuged at 5,625 *g* for 10 min, and the supernatant was collected. The precipitate was re-extracted two additional times with 20 mL of solvent each time, and the supernatants were combined. The supernatant was reduced to dryness under vacuum with a rotary evaporator at 38°C, and the residue was re-dissolved in 2 mL of 100% methanol. This was dried under a stream of N_2_ at 38°C and re-dissolved in 500 μl of 70% (v/v) HPLC-grade methanol diluted with HPLC-grade water. Samples were re-filtered and 15 μl of extract was immediately injected in the HPLC system.

### HPLC analysis and TG determination

A HP1100 high-performance liquid chromatography (HPLC) system was used (Agilent Technologies Inc., Palo Alto, CA) with gradient elution and a μBondapak C18 reversed phase column (10 μm, 4.6 mm×150 mm) (Waters Inc., Milford, MA). The binary gradient employed the mobile phases: (A) phosphate buffer (10.3 mM KH_2_PO_4_ at pH 5.8) and (B) CH_3_CN with a flow rate of 1.2 ml/min according to the following profile adapted from Lim W et al. [30]: 0–20 min, 84–82% A and 16–18% B; 20–60 min, 82–60% A and 18–40% B, 60–120 min 60%–5% A and 40%–95% B. The UV diode array detector was set at 203 nm. Ginsenoside standards included Rg1, Re, Rf, and Rd (National Institutes for Food and Drug Control, NIFDC). Qualitative identification of ginsenoside peaks was determined by cochromatography (equivalent retention time) with chemically pure standards, and quantification was based on the integration of the peak area compared with a standard curve. Results are reported as percent ginsenoside on a dry weight basis.

The spectrophotometric method was used to determine the TG content (mg/g) present in each population’s roots [31]. Each sample extract (50 μl) was diluted to 0.5 ml methanol and reacted at 60°C for 10 min with 8% vanillin solution (0.5 ml) and 87% sulfuric acid (5 ml). The absorbance of the reaction mixture was read at 544 nm against a blank solution.

### Microbial flora analyses

Viable total counts of cultivable bacteria, fungi, actinomycetes, abiogenous Azotobacter, cellulose-decomposing microorganisms, nitrifying bacteria, sulfur bacteria, ammonifying bacteria, and potassium bacteria were determined as colony forming units (CFUs) on agar plates by dilution plate methods. The medium used for the enumeration of bacteria, fungi, actinomycetes, abiogenous Azotobacter, cellulose-decomposing microorganisms, nitrifying bacteria, sulfur bacteria, ammonifying bacteria, and potassium bacteria were beef extract peptone medium, improved Gause’s No.1 medium, Rose bengal medium, Ashby nitrogen free medium, cellulose-Congo red medium, nitrifying bacteria medium, sulfur bacteria medium, peptone ammonifying culture medium, and potassium aluminum silicate agar medium, respectively [32].

### Determination of rhizosphere soil

The activities of sucrase, urease, acid phosphatase, catalase and cellulase in *P*. *ginseng* rhizosphere soil were determined according to Guan [33]. Chemical analyses (total nitrogen, total phosphorus, total potassium, nitrate nitrogen, ammonium nitrogen, available phosphorus, available potassium, and organic matter) were done according to analysis of soil physical and chemical properties [34].

### Data analysis

Amplified bands were scored 1/0 as presence/absence of homologous bands for all samples. The presence/absence data matrix was analyzed using POPGENE version 1.32 [35,36,37] to calculate various genetic diversity parameters, including the percentage of polymorphic loci (Ppl), Shannon’s information index (I) and genetic diversity (h), genetic diversity, gene differentiation coefficient (Gst) and gene flow (Nm), and total genetic diversity (Ht) and within group genetic diversity (Hs). Genetic distance was also generated by POPGENE and a dendrogram was constructed from Nei’s (1978) genetic distance with the unweighted pair-group method of averages (UPGMA) with 1,000 permutations of bootstrapping using MEGA v5.2. SVHFs were computed by the professional software *Similarity Evaluation System for Chromatographic Fingerprint of Traditional Chinese Medicine* (Version 2004 A), which was developed and recommended by Chinese State Food and Drug Administration. This software was also used to synchronize among different samples [38,39]. DPS 14.10 (data processing system) was employed to compute the correlation of SVHF, RIHF, and the contents of Rg1, Re, Rf, and Rd with age, genetic diversity, genetic identity, Shannon (H’) and soil nutrients, stepwise line regression, and path analysis [40].

## Results and discussion

### ISSR profile and genetic analysis

The twelve selected ISSR primers generated 1856 clear and repeatable DNA fragments from eight CPG populations. The amplified DNA fragments ranged from 180 to 2,200 bp in size. DNA fragments of the same size were considered as the same band. In total, 232 ISSR bands were detected with repeatability across 126 *P*. *ginsengs* from eight cultivated populations. The number of bands per primer varied between 14 (UBC836) and 26 (UBC807), with an average of 23.2 (Table 1). Four of 12 primers revealed ISSR loci with 100% polymorphism at the species level, while other primers detected polymorphic loci from 76.9% (primer UBC815) to 94.7% (primer UBC823), leading to an average of 21.2 polymorphic loci per primer (Table 2).

A high level of genetic variation was detected using ISSR markers, with 91.38% polymorphic loci at the species level. The CGS IV population had the highest diversity (*h* = 0.1749, *I* = 0.2595, and *Ppl* = 49.57), while the CGS I population shown the lowest diversity (*h* = 0.0938, I = 0.1409, and *Ppl* = 28.88%) (Table 3). This study revealed that the species-level genetic diversity (*Ppl* = 91.83%, *h* = 0.2454, *I* = 0.3823) in GGS and CGS was higher than that in its cultivated conspecifics (*Ppl* = 85.42%, *h* = 0.2294, *I* = 0.3590) or its cultivated congeneric counterparts, e.g. *P*. *quinquefolius* L. (RAPD: *Ppl* = 45.7%; Allozyme: *Ppl* = 62.5%) and *P*. *notoginseng* (RAPD: *Npl* = 75.5%), and approximated its wild conspecifics (AFLP: *Npl* = 94.4%, *h* = 0.3246) [4,41,42,43,44]. Therefore, genetic diversity in the eight populations (CGS and GGS) selected in this study could represent CPG genetic diversity.

**Table 3:** Genetic diversity within populations of cultivated *P*. *ginseng* based on inter-simple sequence repeat data.

Population	Sample size	Na^a^	Ne^b^	*h*^c^	*I*^d^	Npl^e^	Ppl(%)^f^
CGS IV^g^	15	1.4957	1.3056	0.1749	0.2595	115	49.57
CGS III	16	1.3319	1.1924	0.1138	0.1708	77	33.19
CGS II	15	1.3707	1.2166	0.1241	0.185	86	37.07
CGS I	16	1.2888	1.1607	0.0938	0.1409	67	28.88
GGS I	16	1.4095	1.2239	0.1301	0.196	95	40.95
GGS II	16	1.3578	1.2162	0.1244	0.1847	83	35.78
GGS III	16	1.4698	1.2637	0.1542	0.232	109	46.98
GGS IV	16	1.4526	1.2798	0.1586	0.2344	105	45.26
Mean value		1.3971	1.2324	0.1342	0.2004	92.13	39.71
Species level	126	1.9138	1.3961	0.2454	0.3823	212	91.38

^a^Na: Observed number of alleles

^b^Ne: Effective number of alleles (Kimura and Crow, 1964)

^c^*h*: Nei’s (1973) gene diversity

^d^*I*: Shannon’s Information index (Lewontin, 1972)

^e^Npl: Number of polymorphic loci

^f^Ppl: Percentage of polymorphic loci

^g^Ginseng age

At the species level, the coefficient of gene differentiation (Gst) was 0.4551, and the limited into population gene flow (Nm) was 0.5987 (Table 4). The estimate of the total genetic diversity (Ht) was 0.2463, and the within group genetic diversity (Hs) was 0.1342, indicating that the total genetic diversity in this species (about 55.5%) was primarily from genetic divergence between horticultural *P*. *ginseng* populations. This result indicates that the high genetic diversity in CGS and GGS could be attributed to the dominance of selfing (ranging from 58.14% to 89%) in *P*. *ginseng* [45,46]. Therefore, the genetic identity and diversity of CPG populations are relatively stable and the interference by other populations is relatively small.

**Table 4:** The coefficient of gene differentiation and gene flow at the species level of cultivated *P*. *ginseng*.

Population	Sample size	Ht	Hs	Gst	Nm
Species level	126	0.2463	0.1342	0.4551	0.5987

Ht: total genetic diversity; Hs: genetic diversity; Gst: gene differentiation coefficient; Nm: gene flow.

**Fig 1:**
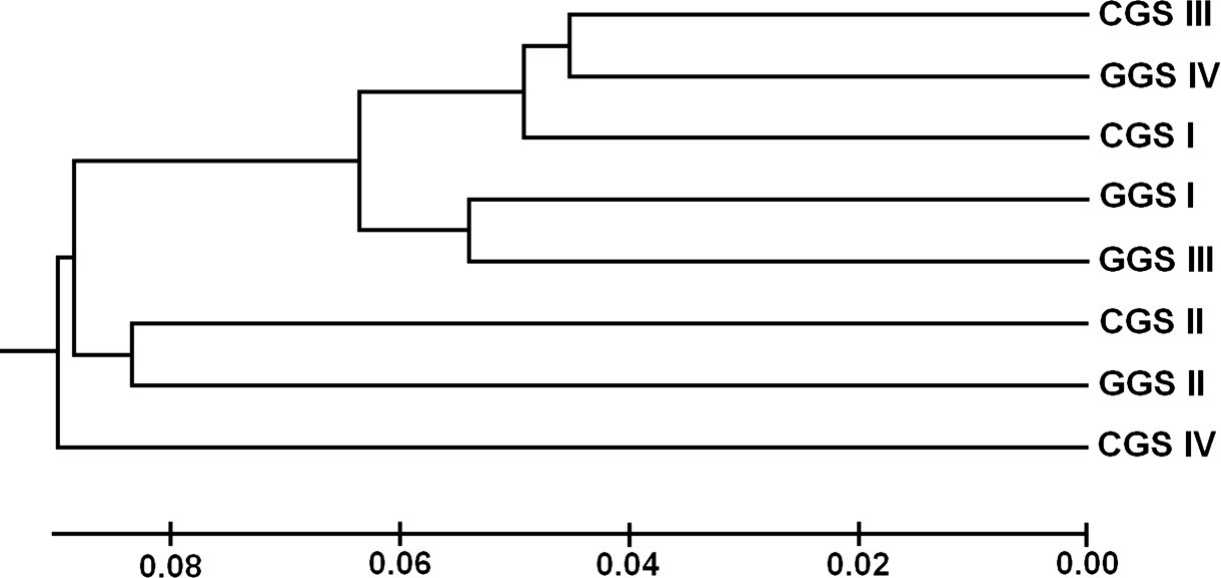
UPGMA dendrogram of garden ginseng (GGS) and cropland ginseng (CGS). UPGMA dendrogram based on 232 inter-simple sequence repeat (ISSR) loci showing the genetic relationships among eight populations of the two main cultivated *P*. *ginseng* (CGS and GGS).

Nei’s (1978) genetic distances ranged from 0.0903 (GGS IV vs. CGS III) to 0.2003 (GGS IV vs. CGS II), with an average of 0.1521 (Table 5). Accordingly, the genetic identity ranged from 0.8001 (CGS I vs. CGS II) to 0.9137 (GGS IV vs. CGS III). The genetic identity (from 0.8416 to 0.8997) of GGS was more uniform than CGS (from 0.8001 to 0.9023). The UPGMA cluster analysis clustered all eight cultivated populations into four groups (Fig 1), rather than the eight cultivated populations attached to two cultivated groups (CGS and GGS). In other words, all populations that belonged to the same cultivated type (GGS and CGS) were not clustered together. This is consistent with a randomly chosen *P*. *ginseng* seed when sown.

**Table 5:** Nei's unbiased measures of genetic identity and genetic distance in garden ginseng (GGS) and cropland ginseng (CGS).

Population	CGS IV	CGS III	CGS II	CGS I	GGS I	GGS II	GGS III	GGS IV
CGS IV	****	0.8541	0.8352	0.8266	0.8424	0.8285	0.8323	0.8297
CGS III	0.1577	****	0.8522	0.9023	0.8810	0.8415	0.8776	0.9137
CGS II	0.1800	0.1599	****	0.8001	0.8324	0.8463	0.8493	0.8185
CGS I	0.1904	0.1028	0.2231	****	0.8896	0.8194	0.8712	0.9104
GGS I	0.1715	0.1266	0.1835	0.1169	****	0.8651	0.8977	0.8776
GGS II	0.1881	0.1726	0.1669	0.1992	0.1449	****	0.8598	0.8416
GGS III	0.1835	0.1305	0.1634	0.1379	0.1079	0.1510	****	0.8875
GGS IV	0.1866	0.0903	0.2003	0.0939	0.1306	0.1724	0.1194	****

### HPLC fingerprint analysis and ginsenoside content

Standard solutions of Rg1, Re, Rf, and Rd were prepared in 70% (v/v) HPLC-grade methanol diluted with HPLC-grade water at final concentrations of 0.03, 0.06, 0.13, 0.25, 0.50, and 1.00 mg/mL, respectively. Calibration was performed by analyzing the four reference solutions in duplicate at six concentration levels, and then the calibration curves were constructed by plotting the peak areas versus the injection concentrations of each compound.

**Fig 2:**
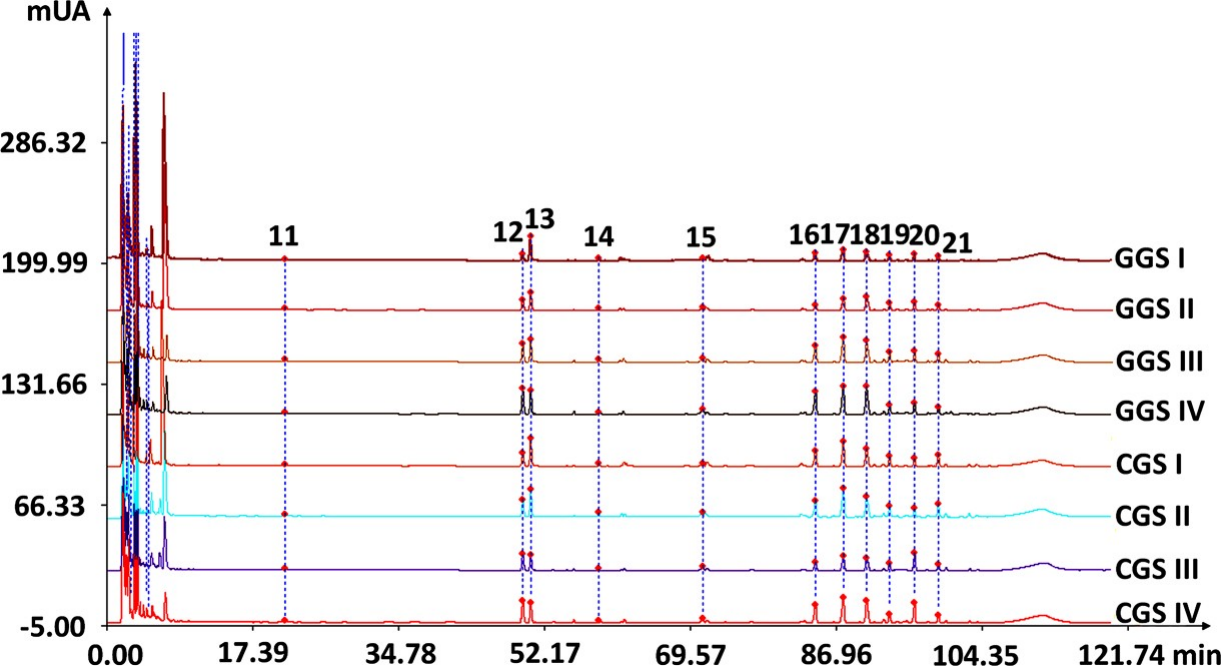
HPLC fingerprints of garden ginseng (GGS) and cropland ginseng (CGS). HPLC fingerprints obtained from batches of eight cultivated *P*. *ginseng* populations. The reference fingerprint was defined as the CGS IV HPLC fingerprint. 1–21 correspond to 21 common peaks, while peaks 1–10 were not intense and not marked. Peaks 12, 13, 15, and 20 were identified as Rg1, Re, Rf, and Rd, respectively.

**Fig 3:**
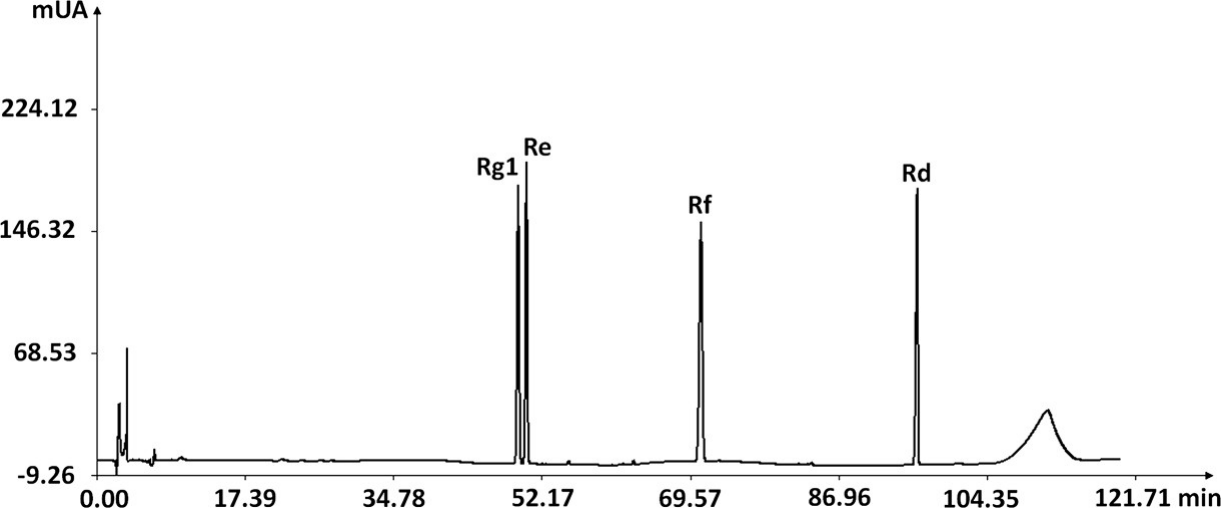
Chromatogram of mixed standard compounds (Rg1, Re, Rf, and Rd).

HPLC fingerprints obtained from eight batches of eight CPG populations and the CGS IV HPLC reference fingerprint are given in Fig 2. The SVHFs versus reference fingerprint are tabulated in Table 6. There were 21 common peaks in all eight batches, and common peaks area accounted for over 52% of the overall peaks area. Common peak area increased with age in the GGS and CGS. Peaks 12, 13, 15, and 20 were identified as Rg1, Re, Rf, and Rd by comparison with the corresponding chemical references chromatogram under the same conditions (Fig 3). Each sample was analyzed in duplicate to determine the mean contents (mg/g) of TG and four selected ginsenosides. The results are shown in Table 6.

**Table 6:** HPLC fingerprint analysis and total ginsenoside (TG) content of garden ginseng (GGS) and cropland ginseng (CGS).

Population	SVHF	RIHF	TG (% w/w)	Rg1(% w/w)	Re(% w/w)	Rf(% w/w)	Rd(% w/w)
CGS IV	1.0000	0.2565	2.1172	0.3293	0.2689	0.0737	0.2843
CGS III	0.9710	0.2000	2.0726	0.2507	0.2047	0.0578	0.2693
CGS II	0.8910	0.1913	2.0406	0.2496	0.3650	0.0523	0.1324
CGS I	0.8130	0.1696	1.7066	0.1573	0.2234	0.0485	0.1028
GGS I	0.8560	0.1609	1.6832	0.1048	0.3313	0.0375	0.087
GGS II	0.9120	0.1696	1.9066	0.1517	0.2453	0.0426	0.1069
GGS III	0.9640	0.2348	1.996	0.2828	0.3086	0.0660	0.1545
GGS IV	0.9830	0.2478	2.3534	0.3834	0.3177	0.0835	0.1642

RIHF is calculated by the Monk (1967) index with the formula R = S/N, where S is the number of HPLC peaks for each sample, and N is the number of HPLC peaks for all samples (common peaks were only counted once) [47]. The RIHF indicates the rich degree of chemical components in CPG roots. The computational results are shown Table 6.

The effect of age was not the same for SVHF, RIHF, TG, Rg1, Re, Rf, and Rd. The TG, Rg1, Rf, and Rd contents increased with increasing age in GGS and CGS. Ginseng age was more approximate and SVHF was higher, indicating that the main chemical composition (the peak accounting for over 5% of the total peak area of the peak) was more similar [48,49]. RIHF increased from age I to IV in GGS and CGS, indicating that as ginseng age increased, chemical components were enriched in CPG root. The content of Re also increased with age from II to IV in GGS, but not in CGS, indicating that other factors could affect the content of Re. In general, with the increase of ginseng age, the content and number of CPG root ginsenosides was greater and CGP bioactive value was much better.

### Rhizosphere soil microbial community diversity analysis

**Fig 4:**
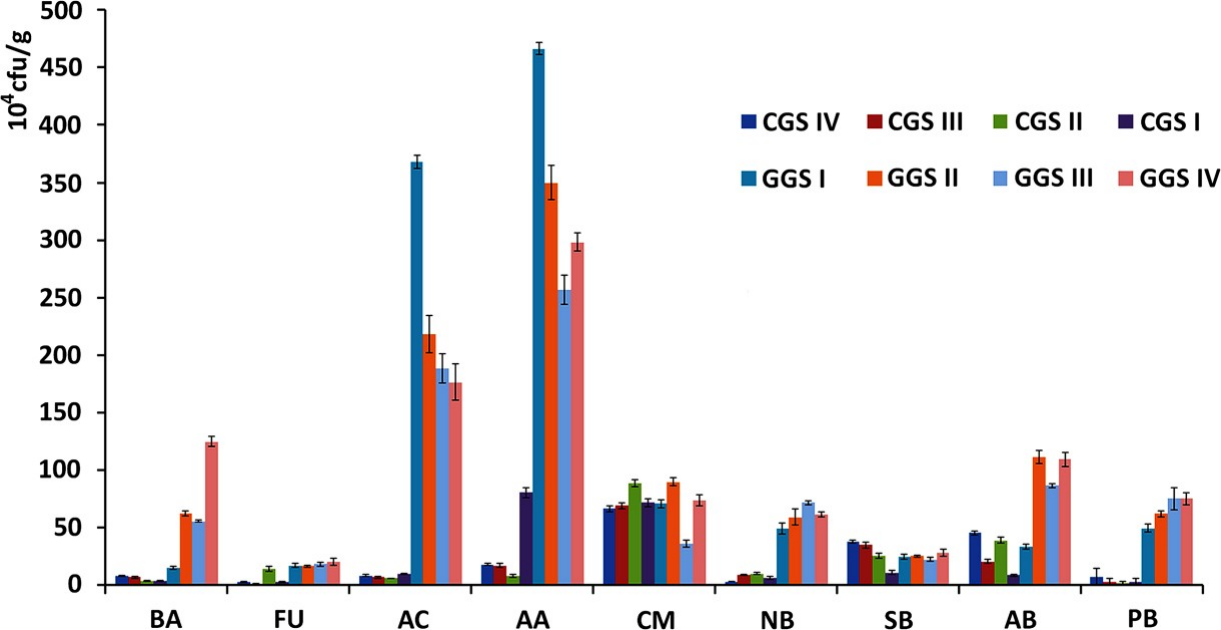
Amounts of rhizosphere soil microorganisms. Bacteria (BA), Fungi (FU), Actinomycetes (AC), Abiogenous Azotobacter (AA), Cellulose-decomposing Microorganisms (CM), Nitrifying Bacteria (NB), Sulfur Bacteria (SB), Ammonifying Bacteria (AB), and Potassium Bacteria (PB) were counted. Eight samples from each of three replicate plots were averaged. Values are mean±standard deviation.

Fig 4 shows the amount of bacteria (BA), fungi (FU), actinomycetes (AC), abiogenous Azotobacter (AA), cellulose-decomposing microorganisms (CM), nitrifying bacteria (NB), sulfur bacteria (SB), ammonifying bacteria (AB), and potassium bacteria (PB) in *P*. *ginseng* rhizosphere soil. The amount of these microorganisms in each age-matched rhizosphere samples was significantly different between CGS and GGS (p<0.01). The microorganisms content in GGS rhizosphere soil was more than five times higher that of the CGS rhizosphere. Diversity and evenness were lower in the CGS than those in GGS, and increased with CGS and GGS age (Table 7). Similar results were also reported by Yong Li et al. and Li Xi-ying et al. [50,51]. Changes in microbial community diversity could be induced by environmental factors, such as overuse of nitrogen, phosphorus fertilizers, and exudates released from roots to their adjacent soil [52,53].

**Table 7:** Culturable microbial community diversity indices for garden ginseng (GGS) and cropland ginseng (CGS).

Sample	Simpson(J)	Shannon(H’)	Evenness(J’)	Brillouin(H)	McIntosh(D_mc_)
CGS IV	0.7878	2.5314	0.7986	2.4198	0.5760
CGS III	0.7642	2.472	0.7798	2.3369	0.5522
CGS II	0.7353	2.3702	0.7477	2.2564	0.5188
CGS I	0.6986	2.1598	0.6813	2.0567	0.4821
GGS I	0.6957	2.1679	0.6839	2.1381	0.4617
GGS II	0.7962	2.6539	0.8372	2.6193	0.5656
GGS III	0.8113	2.7192	0.8578	2.68	0.5849
GGS IV	0.8263	2.7966	0.8822	2.7618	0.6015

### Rhizosphere soil enzymatic activities

Soil enzymes play an important role in the material cycle and energy transformation of soil ecological systems. They are also important for catalyzing reactions necessary for the life of microorganisms and plants, decomposition of organic residues, cycling of nutrients, and formation of organic matter and soil structure [37,54]. Soil enzyme activities may be considered early and sensitive indicators to measuring the degree of soil degradation in both natural and agro-ecosystems, and can be an important indicator of soil fertility [37,55,56,57]. Table 8 shows the rhizosphere soil enzymatic activities of eight populations of GGS and CGS. The enzymatic activities of sucrase, acid phosphatase, and cellulase firstly increased from age I to II, and then decreased from age II to III, and finally increased from age III to IV. In contrast, the urease activity firstly decreased from age I to II, and then increased from age II to III, and finaly decreased from age III to IV. Catalase activity increased with CGS and GGS age. The GGS enzymatic activities of sucrase, acid phosphatase catalase and cellulase were higher than their peers, while the urease activity was the opposite. Therefore, the soil fertility in GGS was higher than in CGS.

**Table 8:** Activities of sucrase, urease, acid phosphatase, catalase, and cellulase in the rhizosphere soil of garden ginseng (GGS) and cropland ginseng (CGS).

Population	Sucrase mg GLU/g·d	Urease mg NH_4_^-1^/g·d	Acid Phosphatase mg PNP/g·d	Catalase mg KMnO_4_/g·d	Cellulase mg GLU/g·d
CGS IV	1.7072	0.5680	1.6530	1.1799	0.2790
CGS III	0.8640	1.0860	1.2800	1.1286	0.1110
CGS II	1.1368	0.8560	1.7070	1.0773	0.2600
CGS I	0.6284	1.0500	1.1070	1.0260	0.1300
GGS I	2.4770	0.8340	2.4250	1.9490	0.1900
GGS II	4.5240	0.5980	2.9080	2.0010	0.2490
GGS III	1.9610	0.9580	2.8130	2.0520	0.1200
GGS IV	3.8040	0.8350	3.1280	2.1030	0.2800

### Rhizosphere soil nutrient analysis

Table 9 shows that the GGS rhizosphere soil exhibited the same available P as CGS, and 2–3 fold greater soil total N, nitrate N, ammonium N, available K, and organic matter than CGS. However, total P and total K in GGS were only approximately 70% as much as in CGS.

**Table 9:** Rhizosphere soil nutrient analysis for eight populations of garden ginseng (GGS) and cropland ginseng (CGS).

Population	CGS IV	CGS III	CGS II	CGS I	GGS I	GGS II	GGS III	GGS IV
Total N %	0.2182	0.2008	0.1387	0.1722	0.4442	0.5298	0.4237	0.4744
Total P %	0.1559	0.1974	0.1157	0.1762	0.1197	0.1501	0.1414	0.1494
Total K %	2.6230	2.9937	2.7992	2.5451	1.8820	2.1202	1.9608	1.9156
Nitrate N g/kg	0.0137	0.0217	0.0154	0.0190	0.0470	0.0663	0.0343	0.1014
Ammonium N g/kg	0.0097	0.0100	0.0110	0.0115	0.0205	0.0204	0.0198	0.0348
available P g/kg	0.0307	0.0565	0.0152	0.0251	0.0368	0.0347	0.0091	0.0411
available K g/kg	0.1442	0.1106	0.0991	0.1036	0.4863	0.2091	0.1964	0.4217
Organic Matter %	3.9015	4.0678	2.4477	3.1277	9.0197	9.4802	8.1740	8.9503

### Statistical analysis

The correlation coefficient of SVHF, RIHF, and the content of TG, Rg1, Re, Rf, and Rd with rhizosphere soil enzymatic activities and nutrients was not significant (p>0.05) (Table 10). The correlation coefficient of the content of TG and Rg1 with age and Shannon (H’) was significant (p<0.05) and positive. The correlation coefficient of SVHF, RIHF, and the content of Rf with age, genetic diversity index (*h*), and Shannon (H') was significant (p<0.05) and positive. The correlation coefficient of Re content with total phosphorus was very significant (p<0.01) and negative. The age and genetic identity were significantly related to the content of Rd (p<0.05). Thus, selection for these significant correlative factors may improve SVHF, RIHFs, TG, and selected four-monomer ginsenoside content (Rg1, Re, Rf, and Rd). The stepwise line regression equations of SVHF, RIHF, TG, and selected four-monomer ginsenoside content as dependent variable (*Y*) with their own significant correlative factors (*X*_*n*_) are shown in Table 11. Path analysis results of SVHF, RIHF, TG, and four-monomer ginsenoside content to their own significant correlative factors are shown in Table 12. Ginseng age was the most important influence on SVHF, RIHF, and TG, Rg1, Rf, and Rd contents, but had no effect on Re content. Ginseng age directly affected TG (0.8187), Rg1 (0.9869), and Rf (0.9996), and was higher than their respective Shannon (H') (microbial community diversity index) (0.043, −0.1080, and −0.1019). Shannon (H') had a direct positive effect on TG content, but a direct negative effect on Rg1 and Rf contents. Determination coefficients of TG, Rg1, and Rf contents were 0.7278, 0.8158, and 0.8486, respectively. This means that the theoretical values calculated through the regression equations of TG, Rg1, and Rf contents had considered their respective variability of 72.78%, 81.58%, and 84.86%. The first direct effect to SVHF was age (0.8336), second was Shannon (H') (0.1170), and the third was *h* (0.0441). The first direct effect to RIHF was age (0.8341), second was *h* (0.2874), and the third was Shannon (H') (−0.1519). The determination coefficients of SVHF and RIHF were 0.9291 and 0.9224, respectively. This means that the theoretical values of SVHF and RIHF had considered their respective variability of 92.91% and 92.24%. Therefore, ginseng age, *h*, and Shannon (H') are more approximate and SVHF was higher, indicating that the main chemical composition is more similar. The same ginseng age, *h*, and Shannon (H’) might produce a similar amount of chemical components in CPG roots. Therefore, increasing age and genetic diversity while reducing microbial community diversity could increase the number of chemical components. For one ginseng population, appropriate selection for age and Shannon (H') could result in increasing RIHF, TG, Rg1, and Rf contents, but had little effect on SVHF. The age (0.5323) direct effect on Rd was higher than genetic identity (0.4511), and its determination coefficient was 0.7330. This indicates that improving age and genetic consistency could increase the content of Rd. Total phosphorus (−0.9249) was a directly negative effect on Re content, and its determination coefficient is 0.8555. Thus adding phosphate fertilizer could decrease the content of Re. This result is the same as Konsler T R. at el. [58].

**Table 10:** Simple correlation of similarity values of HPLC fingerprint (SVHF), richness values of HPLC fingerprint (RIHF), and the contents of total ginsenosides (TG), Rg1, Re, Rf, and Rd with age, genetic diversity, genetic identify, Shannon (H’), and soil nutrients.

	SVHF	RIHF	TG	Rg1	Re	Rf	Rd
Age	0.96**	0.94**	0.91**	0.94**	0.00	0.92**	0.76*
*h*	0.77*	0.85**	0.63	0.61	0.40	0.72*	0.45
Genetic identity	0.49	0.54	0.25	0.37	-0.13	0.39	0.72*
Shannon(H’)	0.80**	0.69*	0.78*	0.70*	0.06	0.69*	0.32
Sucrase	0.23	0.05	0.24	0.01	0.17	0.07	-0.30
Urease	-0.28	-0.21	-0.18	-0.06	-0.20	-0.08	-0.07
Acid Phosphatase	0.29	0.21	0.29	0.14	0.44	0.20	-0.35
Catalase	0.20	0.10	0.11	0.00	0.33	0.09	-0.39
Cellulase	0.27	0.30	0.47	0.32	0.46	0.30	0.03
Total nitrogen	0.15	0.00	0.05	-0.10	0.17	-0.01	-0.39
Total phosphorus	0.20	0.09	0.11	0.17	-0.92**	0.19	0.53
Total potassium	0.04	-0.03	0.09	0.13	-0.39	-0.03	0.55
Nitrate nitrogen	0.20	0.13	0.38	0.19	0.20	0.27	-0.31
Ammonium nitrogen	0.20	0.22	0.37	0.25	0.36	0.34	-0.35
Available phosphorus	0.23	-0.08	0.19	0.05	-0.51	0.03	0.39
Available potassium	-0.01	-0.02	0.00	-0.09	0.44	0.02	-0.37
Organic Matter	0.14	-0.02	0.02	-0.12	0.20	-0.02	-0.39

*p<0.05

**p<0.01

**Table 11:** Regression equations of similarity values of HPLC fingerprint (SVHF), richness values of HPLC fingerprint (RIHF), and the contents of total ginsenosides (TG), Rg1, Re, Rf, and Rd with their own significance factors.

	Standard Equations	Regression coefficient
SVHF	*Y*_*sv*_ = 0.7113+0.0466*X*_*1*_+0.1112*X*_*2*_+0.0326*X*_3_**	0.9639
RIHF	*Y*_*ri*_ = 0.1423+0.02643*X*_*1*_+0.4104*X*_*2*_-0.0240*X*_3_*	0.9604
TG	*Y*_*TG*_ = 1.5914+0.1327*X*_*1*_+0.0348*X*_3_*	0.8531
Rg1	*Y*_*Rg1*_ = 0.1508+0.0758*X*_*1*_-0.0381*X*_3_*	0.9032
Re	*Y*_*Re*_ = 0.5732–1.9244*X*_4_**	0.9249
Rf	*Y*_*Rf*_ = 0.0414+0.0132*X*_*1*_-0.0067*X*_3_**	0.9212
Rd	*Y*_*Rd*_-0.4145+0.0335*X*_*1*_*+*0.5764*X*_5_*	0.8561

*X*_*1*_: Age, *X*_*2*_: *h*, *X*_*3*_: Shannon (H'), *X*_*4*_: Total phosphorus, *X*_*5*_: Genetic identity

*p<0.05

**p<0.01

**Table 12:** Path analyses of similarity values of HPLC fingerprint (SVHF), richness values of HPLC fingerprint (RIHF), and the contents of total ginsenosides (TG), Rg1, Re, Rf, and Rd with their own significance factors.

	Factor	Direct effect	Indirect effect				Determination coefficient (R_d_)	Remaining path coefficient (Rr)
			*X*_*1*_	*X*_*2*_	*X*_*3*_	*X*_*5*_		
SVHF	*X*_*1*_	0.8336	-	0.0347	0.0924	-	0.9291	0.2664
	*X*_*2*_	0.0441	0.6553	-	0.0748			
	*X*_*3*_	0.1170	0.6584	0.0282	-	-		
RIHF	*X*_*1*_	0.8341	-	0.2259	-0.1199	-	0.9224	0.2785
	*X*_*2*_	0.2874	0.6557	-	-0.097			
	*X*_*3*_	-0.1519	0.6588	0.1836	-	-		
TG	*X*_*1*_	0.8187	-	-	0.034	-	0.7278	0.5218
	*X*_*3*_	0.043	0.6466	-	-	-		
Rg1	*X*_*1*_	0.9869	-	-	-0.086	-	0.8158	0.4292
	*X*_*3*_	-0.1080	0.7858	-	-	-		
Re	*X*_*4*_	-0.9249	-	-	-	-	0.8555	0.3801
Rf	*X*_*1*_	0.9996	-	-	-0.0805	-	0.8486	0.3891
	*X*_*3*_	-0.1019	0.7894	-	-	-		
Rd	*X*_*1*_	0.5323	-	-	-	0.2313	0.7330	0.5845
	*X*_*5*_	0.4511	0.2729	-	-	-		

*X*_*1*_: Age, *X*_*2*_: *h*, *X*_*3*_: Shannon (H'), *X*_*4*_: Total phosphorus, *X*_*5*_: Genetic identity

During cultivation of *P*. *ginseng*, appropriate selection for various factors could improve SVFH, RIHF, and the content of ginsenosides (TG, Rg1, Re, Rf, and Rd). For example, appropriate age increases could improve RIHF and TG, Rg1, Rf, and Rd contents in CPG root. However, rhizosphere soil microbial community diversity increased with age (Tables 7 and 13). This could be due to secretions from the ginseng root causing increases in specific carbon substrates and/or signaling compounds supporting increased rhizosphere microbial community diversity [59]. Because increasing rhizosphere soil microbial community diversity could decrease RIHF and TG, Rg1, and Rf contents according to stepwise line regression equations and path analyses, appropriate management measures can be taken to reduce microbial community diversity (Shannon [H']) while managing CPG. The simple correlation of Shannon (H') with age, genetic diversity, genetic identify, and soil nutrients is shown in Table 13. The moderate correlation (0.5≤|correlation coefficient|<0.8) of Shannon (H') with sucrase, acid phosphatase, catalase, total nitrogen, nitrate nitrogen, and ammonium nitrogen was positive, while the correlation of nitrate nitrogen and ammonium nitrogen with acid Phosphatase, catalase and total nitrogen was positive and significant (p<0.05). The simple correlation of nitrate nitrogen and ammonium nitrogen with RIHF and the content of TG, Rg1, and Rf was not correlated (|correlation coefficient|<0.3) or had low correlation (0.3≤|correlation coefficient|<0.5). Thus, appropriate reduction in the amount of ammonium nitrogen and nitrate nitrogen in fields could reduce Shannon (H') and improve RIHF and TG, Rg1, and Rf contents. This conjecture was consistent with published results that root N is negatively correlated with root Rg1 and the accumulation of TG was severely inhibited when NH_4_^+^ content is increased [58,60].

**Table 13:** Simple correlation of Shannon (H') with age, genetic diversity, genetic identify, and soil nutrients.

Correlation coefficient	Shannon (H')	Nitrate nitrogen	Ammonium nitrogen
Shannon (H')	1.00	0.56	0.55
age	0.79*	0.24	0.25
genetic diversity	0.64	0.26	0.36
genetic identity	0.05	-0.39	-0.4
Sucrase	0.59	0.86**	0.77*
Urease	-0.31	-0.23	-0.14
Acid Phosphatase	0.67	0.83**	0.87**
Catalase	0.54	0.80**	0.85**
Cellulase	0.31	0.39	0.32
Total nitrogen	0.51	0.82**	0.81**
Total phosphorus	0.06	-0.16	-0.28
Total potassium	-0.31	-0.72*	-0.82**
Nitrate nitrogen	0.56	1.00	0.96**
Ammonium nitrogen	0.55	0.96**	1.00
Available phosphorus	0.01	0.29	0.11
Available potassium	0.08	0.73*	0.79*
Organic Matter	0.46	0.80**	0.81**

*p<0.05

**p<0.01

In this study, we defined the quantitative relationship between SVHF, RIHF, and the contents of Rg1, Re, Rf, and Rd and their respective significant correlative factors (age, genetic diversity, genetic identify, Shannon [H’], and soil nutrients). These findings could help progress CPG cultivation methods. The regression coefficients of acquired regression equations were less than 0.999 and remaining path coefficients were also larger (>0.2664) (Tables 11 and 12), indicating that some factors influencing SVHF, RIHF, and TG, Rg1, Re, Rf, and Rd contents were not taken into account. These factors might include light, rainfall, moisture, temperature, uncultivable microbial community, soil physical properties, soil chemical properties, soil trace elements (such as Mn, Me, and Zn), and cultural practices. If these factors influencing the content and constituents of CPG could be controlled, an accurate quantitative relationship between chemical content and factors could be determined by mathematical analysis. These accurate quantitative relationships combined with modern networks and automatic detection technology can establish the best CPG cultivation methods. These methods allow us to control the content and constituents of CPG bioactive ingredients by adjusting the related influencing factors. Because the potential benefits of specific ginsenosides on cancer and diabetes has been published [61,62], CPG cultivation methods enhancing the production of specific monomer ginsenosides and other bioactive ingredients could seriously impact commerce in this medicinal herb and its future role in public health.

## Conclusions

In conclusion, we obtained the regression equations of similarity values of HPLC fingerprint (SVHF), richness index of HPLC fingerprint (RIHF) and the TG, Rg1, Re, Rf, and Rd contents with their respective significant correlation factors. SVHF and RIHF were influenced not only by age and microbial community diversity but also genetic diversity. For SVHF, the relative importance is age>microbial community diversity>genetic diversity. For RIHF, the relative importance is age>genetic diversity>microbial community diversity. The factors that influence TG, Rg1, and Rf content were ginseng age and microbial community diversity, by contrast, ginseng age was the main influencing factor. Ginseng age and genetic identity influenced Rd content, and age was more important. Re was influenced only by total phosphorus. Therefore, under the same climate, the relative importance of genes, age, and soil factors were not the same for SVHF, RIHF, and TG Rg1, Re, Rf, and Rd contents in CPG. In general, increasing age and decreasing Shannon (H') could improve RIHF and TG, Rg1 Rf, and Rd contents, but had little effect on SVHF; increasing age and genetic diversity identity could also improve the content of Rd; appropriate decreases in total phosphorus might increase the content of Re. These findings can help progress CPG cultivation methods, which could help achieve customized CPG bioactive ingredients through regulating genotypes and cultural conditions.

## Supporting information

S1 Table Basic climatic information of Ji 'an, Jilin Province, China (according to the data from 1971–2000). (DOCX)

## References

[1] Chinese Academy of Science. Editorial Committee of the Flora of China: *Flora of China*. Volume 54 Beijing: Science Press; 1978 pp. 180.

[2] Wen J, Zimmer EA. Phylogeny and biogeography of *Panax*L. (the ginseng genus, Araliaceae): inferences from ITS sequences of nuclear ribosomal DNA. Mol Phylogenet Evol. 1996;6(2): 167–177. 10.1006/mpev.1996.0069 8899721

[3] Lee JW, Kim YC, Jo IH, Seo AY, Lee JH, Kim OT, et al Development of an ISSR-Derived SCAR Marker in Korean Ginseng Cultivars (*Panax ginseng* C. A. Meyer). J. Ginseng Res. 2011;35(1): 52–59.

[4] Li S, Li J, Yang XL, Cheng Z, Zhang WJ. Genetic diversity and differentiation of cultivated P. ginseng (*Panax ginseng* C. A. Meyer) populations in North-east China revealed by inter-simple sequence repeat (ISSR). Genet Resour Crop Evol. 2011;58: 815–824.

[5] Wang CZ, Ni M, Sun S, Li XL, He H, Mehendale SR, et al Detection of adulteration of notoginseng root extract with other panax species by quantitative HPLC coupled with PCA. J Agric Food Chem. 2009;57(6): 2363–2367. 10.1021/jf803320d 19256509 PMC2705280

[6] Zuo G, Guan T, Chen DL, Li CL, Jiang R, Luo CY, et al Total saponins of *Panax ginseng* induces K562 cell differentiation by promoting internalization of the erythropoietin receptor. Am J Chin Med. 2009;37(4): 747–57. 10.1142/S0192415X09007211 19655412

[7] Taira S, Ikeda R, Yokota N, Osaka I, Sakamoto M, Kato M, et al Mass spectrometric imaging of ginsenosides localization in *Panax ginseng* root. Am J Chin Med. 2010;38(3): 485–93. 10.1142/S0192415X10008007 20503467

[8] Schlag EM, Mclntosh MS. Ginsenoside content and variation among and within American ginseng (*Panax quinquefolius*L.) populations. Phytochemistry. 2006;67: 1510–1519. 10.1016/j.phytochem.2006.05.028 16839573

[9] Leung KW, Wong AST. Pharmacology of ginsenosides: a literature review. Chinese Medicine. 2010;5(20): 1–7. 20537195 10.1186/1749-8546-5-20 PMC2893180

[10] Kampen JV, Robertson H, Hagg T, Drobitch R. Neuroprotective actions of the ginseng extract G115 in two rodent models of Parkinson's disease. Experimental neurology. 2003;184(1): 521–529. 10.1016/j.expneurol.2003.08.002 14637121

[11] Liao B, Newmark H, Zhou R. Neuroprotective Effects of Ginseng Total Saponin and Ginsenosides Rb1 and Rg1 on Spinal Cord Neurons *in Vitro*. Experimental neurology. 2002;173(2): 224–234. 10.1006/exnr.2001.7841 11822886

[12] Lim JH, Wen TC, Matsuda S, Tanaka J, Maeda N, Peng H, et al Protection of ischemic hippocampal neurons by ginsenoside Rb1, a main ingredient of ginseng root. Neuroscience research. 1997;28(3): 191–200. 10.1016/s0168-0102(97)00041-2 9237267

[13] Cho J, Park W, Lee S, Ahn W, Lee Y. Ginsenoside-Rb1 from *Panax ginseng C*.*A*. *Meyer* activates estrogen receptor-α and-β, independent of ligand binding. Journal of Clinical Endocrinology & Metabolism. 2004;89(7): 3510–3515. 10.1210/jc.2003-031823 15240639

[14] Cheng Y, Shen LH, Zhang JT. Anti-amnestic and anti-aging effects of ginsenoside Rg1 and Rb1 and its mechanism of action. ACTA pharmacologica sinica. 2005;26(2): 143–149. 10.1111/j.1745-7254.2005.00034.x 15663889

[15] Kenarova B, Neychev H, Hadjiivanova C, Petkov VD. Immunomodulating activity of ginsenoside Rg1 from *Panax ginseng*. Japanese journal of pharmacology. 1990;54(4): 447–449. 10.1254/jjp.54.447 2087006

[16] Liu M, Zhang JT. Studies on the mechanisms of immunoregulatory effects of ginsenoside Rg1 in aged rats. Acta pharmaceutica Sinica. 1995;31(2): 95–100. 8762468

[17] Zhou W, Chai H, Lin PH, Lumsden AB, Yao QZ, Chen CY. Ginsenoside Rb1 blocks homocysteine-induced endothelial dysfunction in porcine coronary arteries. Journal of vascular surgery. 2005;41(5): 861–868. 10.1016/j.jvs.2005.01.054 15886672

[18] Zhang QH, Wu CF, Duan L, Yang JY. Protective effects of total saponins from stem and leaf of *Panax ginseng* against cyclophosphamide-induced genotoxicity and apoptosis in mouse bone marrow cells and peripheral lymphocyte cells. Food and Chemical Toxicology. 2008;46(1): 293–302. 10.1016/j.fct.2007.08.025 17904265

[19] Liu WK, Xu SX, Che CT. Anti-proliferative effect of ginseng saponins on human prostate cancer cell line. Life sciences. 2000;67(11): 1297–1306. 10.1016/s0024-3205(00)00720-7 10972198

[20] Hofseth LJ, Wargovich MJ. Inflammation, cancer, and targets of ginseng. The Journal of nutrition. 2007;137(1): 183–185. 10.1093/jn/137.1.183S 17182823

[21] Choi YJ, Lee HJ, Kang DW, Han IH, Choi BK, Cho WH. Ginsenoside Rg3 induces apoptosis in the U87MG human glioblastoma cell line through the MEK signaling pathway and reactive oxygen species. Oncology Reports. 2013;30(3) 1362–1370 10.3892/or.2013.2555 23783960

[22] Ling C, Li Y, Zhu X, Zhang C, Li M. Ginsenosides may reverse the dexamethasone-induced down-regulation of glucocorticoid receptor. General and comparative endocrinology. 2005;140(3): 203–209. 10.1016/j.ygcen.2004.11.003 15639148

[23] Nocerino E, Amato M, Izzo AA. The aphrodisiac and adaptogenic properties of ginseng. Fitoterapia. 2000;71: 1–5. 10930706 10.1016/s0367-326x(00)00170-2

[24] Nah SY, Kim DH, Rhim H. Ginsenosides are any of them candidates for drugs acting on the central nervous system? CNS Drug Rev. 2007;13: 381–404. 10.1111/j.1527-3458.2007.00023.x 18078425 PMC6494168

[25] Nah SY, Bhatia KS, Lyles J, Ellinwood EH, Lee TH. Effects of ginseng saponin on acute cocaine-induced alterations in evoked dopamine release and uptake in rat brain nucleus accumbens. Brain Res. 2009;1248: 184–90. 10.1016/j.brainres.2008.10.064 19026615 PMC2667959

[26] Chen CF, Chiou WF, Zhang JT. Comparison of the pharmacological effects of Panax ginseng and *Panax quinquefolium*. Acta Pharmacol Sinica. 2008;29: 1103–8. 10.1111/j.1745-7254.2008.00868.x 18718179

[27] Sengupta S, Toh SA, Sellers LA, Skepper JN, Koolwijk P, Leunq HW, et al Modulating angiogenesis: the yin and the yang in ginseng. Circulation. 2004;110: 1219–25. 10.1161/01.CIR.0000140676.88412.CF 15337705

[28] Yue PY, Mak NK, Cheng YK, Leung KW, Ng TB, Fan DT, et al Pharmacogenomics and the Yin/Yang actions ofginseng: anti-tumor, angiomodulating and steroid-like activities of ginsenosides. Chin. Med. 2007;2(6): 1–21. 17502003 10.1186/1749-8546-2-6 PMC1876803

[29] Davy CW Lee, Allan SY Lau. Effects of *Panax ginsengon* Tumor Necrosis Factor-α-Mediated Inflammation: A Mini-Review. Molecules. 2011;16: 2802–2816. 10.3390/molecules16042802 21455094 PMC6260618

[30] Lim W, Mudge KW, Vermeylen F. Effects of population, age, and cultivation methods on ginsenoside content of wild American ginseng (*Panax quinquefolium*). Journal of agricultural and food chemistry. 2005;53(22): 8498–8505. 10.1021/jf051070y 16248544

[31] Lui JH, Staba EJ. The ginsenosides of various ginseng plants and selected products. Journal of Natural Products. 1980;43(3): 340–346.

[32] Xu GH, Zheng HY. Handbook of Analysis Methods of Soil Microbiology. Beijing: Agricultural Press; 1986 pp. 102.

[33] Guan SY. Methods for Determination of Soil Enzyme Activity. Beijing: Agricultural Press;1986 pp. 234.

[34] Nanjing Institute of Soil Science, CAS. Analysis of soil physical and chemical properties. Shanghai: Shanghai Sci.& Tech. Press; 1981 pp. 134.

[35] Yeh FC, Yang RC, Boyle T; 1999 POPGENE, version 1.32: the user friendly software for population genetic analysis. Molecular Biology and Biotechnology Centre, University of Alberta, Edmonton, AB, Canada.

[36] Francis CY, Yang RC; 2000. Popgene version 1.32. Available from: http://www.seekbio.com/DownloadShow.asp?id=1059

[37] Doelman P, Haanstra L. Short-and long-term effects of heavy metals on phosphatase activity in soils: An ecological dose-response model approach. Biology and Fertility of Soils. 1989;8(3): 235–241.

[38] Tistaert C, Dejaegher B, Heyden YV. Chromatographic separation techniques and data handling methods for herbal fingerprints: a review. Analytica chimica acta. 2011;690(2): 148–161. 10.1016/j.aca.2011.02.023 21435470

[39] Chen Y, Yan Y, Xie MY, Nie SP, Liu W, Gong xf, et al Development of a chromatographic fingerprint for the chloroform extracts of *Ganoderma lucidum* by HPLC and LC–MS. Journal of pharmaceutical and biomedical analysis. 2008;47(3): 469–477. 10.1016/j.jpba.2008.01.039 18337046

[40] Tang QY, Feng MG DPS data processing system: experimental design, statistical analysis and data mining. Science, Beijing. 2007 10.1111/j.1744-7917.2012.01519.x 23955865

[41] Bai D, Brandle J, Reeleder R. Genetic diversity in North American ginseng (*Panax quinquefolius* L.) grown in Ontario detected by RAPD analysis. Genome. 1997;40(1): 111–115. 10.1139/g97-015 18464811

[42] Cruse-Sanders JM, Hamrick JL. Genetic diversity in harvested and protected populations of wild American ginseng, *Panax quinquefolius* L.(Araliaceae). American Journal of Botany. 2004;91(4): 540–548. 10.3732/ajb.91.4.540 21653409

[43] Li S, Li J, Yang XL, Cheng Z, Zhang WJ. Genetic diversity and differentiation of cultivated P. ginseng (*Panax ginseng* CA Meyer) populations in North-east China revealed by inter-simple sequence repeat (ISSR) markers. Genetic Resources and Crop Evolution. 2011;58(6): 815–824.

[44] Reunova GD, Kats IL, Muzarok TI, Zhuravlev YN. Polymorphism of RAPD, ISSR and AFLP markers of the *Panax ginseng* CA Meyer (Araliaceae) genome. Russian Journal of Genetics. 2010;46(8): 938–947. 20873202

[45] Zhao Y, Gu X, Wu L. Researches on categories, characteristics, and utilization value of cultivated P. ginseng germplasm resources. Chinese Traditional and Herbal Drugs. 2007;38(2): 294.

[46] Culley TM, Wallace LE, Gengler-Nowak KM, Crawford DJ. A comparison of two methods of calculating GST, a genetic measure of population differentiation. American Journal of Botany. 2002;89(3): 460–465. 10.3732/ajb.89.3.460 21665642

[47] Monk CD. Tree species diversity in the eastern deciduous forest with particular reference to north central Florida. Am. Natur. 1967;101: 173–87

[48] Nanping Z, Xinyue X, Ping Z. Study on the establishing of reference fingerprint for the traditional Chinese medicine. Chinese Pharmaceutical Affairs. 2003;6: 347–350.

[49] Qinglei S, Yunliang L, He Z. Study on the Similarity Between HPLC Fingerprints. Chemical Analysis and Meterage. 2006;15(6): 54–55.

[50] Li Y, Ying YX, Zhao DY, Ding WL. Microbial community diversity analysis of *Panax ginseng* rhizosphere and non-rhizosphere soil using randomly amplified polymorphic DNA method. Open Journal of Genetics. 2012;2: 95–102.

[51] Li XY, Jin HQ, Jia B. Biodiversity of soil microorganisms in the fields of different growing years of Ginseng. Journal of Agricuhural Science Yanbian University. 2011;33(2): 133–136.

[52] Yao HY, Jiao XD, Wu FZ. Effects of continuous cucumber cropping and alternative rotations under protected cultivation on soil microbial community diversity. Plant Soil. 2006;284: 195–203.

[53] Lin XG, Yin R, Zhang HY, Huang JF, Chen RR, Cao ZH. Changes of soil microbiological properties caused by land use changing from rice-wheat rotation to vegetable cultivation. Environmental Geochemistry and Health. 2004;26: 119–128. 15499767 10.1023/b:egah.0000039574.99651.65

[54] Balota EL, Kanashiro M, Colozzi Filho A, Andrade DS, Dick RP. Soil enzyme activities under long-term tillage and crop rotation systems in subtropical agro-ecosystems. Brazilian Journal of Microbiology. 2004;35(4): 300–306.

[55] Dick RP, Pankhurst C, Doube B M. Soil enzyme activities as integrative indicators of soil health. Biological indicators of soil health. 1997: 121–156.

[56] Piotrowska A, Dlugosz J, Zamorski R, Bogdanowicz P. Changes of enzymatic activity in soil supplemented with microbiological preparation. UGmax®19th World Congress of Soil Science. 2010.

[57] Trasar-Cepeda C, Leiros MC, Seoane S, Gil-Sotres F. Limitations of soil enzymes as indicators of soil pollution. Soil Biology and Biochemistry. 2000;32(13): 1867–1875.

[58] Konsler TR, Zito SW, Shelton JE, Staba EJ. Lime and phosphorus effects on American ginseng: II. Root and leaf ginsenoside content and their relationship. Journal of the American Society for Horticultural Science. 1990;115(4): 575–580.

[59] Broeckling CD, Broz AK, Bergelson J, Manter DK, Vivanco JM. Root exudates regulate soil fungal community composition and diversity. Applied and Environmental Microbiology. 2008;74(3): 738–744. 10.1128/AEM.02188-07 18083870 PMC2227741

[60] Yu KW, Gao WY, Hahn EJ, Paek KY. Effects of macro elements and nitrogen source on adventitious root growth and ginsenoside production in ginseng (*Panax ginseng* CA Meyer). Journal of Plant Biology. 2001;44(4): 179–184.

[61] Murphy L. Effects of *American ginseng* on breast cancer and prostate cancer cells. American Ginseng; Production in the 21st Century; Conference Proceedings 2000; Cornell Cooperative Extension of Green County. 2000: 39–45.

[62] Attele AS, Zhou YP. Antidiabetic effects of *Panax ginseng* berry extract and the identification of an effective component. Diabetes. 2002;51(6): 1851–1858. 10.2337/diabetes.51.6.1851 12031973

