# Supplementary material for: Factors influencing cultivated ginseng (Panax ginseng C. A. Meyer) bioactive compounds
Source: PLoS One. 2019 Oct 16;14(10):e0223763. doi: 10.1371/journal.pone.0223763 (PMC6795439; doi:10.1371/journal.pone.0223763)
Supplement: S1 Table — (DOCX) [file pone.0223763.s001.docx]

|  | **January** | **February** | **March** | **April** | **May** | **June** | **July** | **August** | **September** | **October** | **November** | **September** |
| --- | --- | --- | --- | --- | --- | --- | --- | --- | --- | --- | --- | --- |
| **Mean temperature (℃)** | -12.3 | -7.8 | 0.5 | 9.1 | 15.2 | 20.0 | 23.3 | 22.5 | 16.0 | 8.4 | -0.3 | -8.7 |
| **Mean maximum temperature (℃)** | -5.2 | -0.6 | 6.9 | 16.6 | 22.7 | 26.6 | 28.8 | 28.3 | 23.0 | 15.9 | 5.5 | -2.8 |
| **Extreme maximum temperature (℃)** | 5.0 | 14.0 | 20.1 | 30.4 | 34.5 | 36.3 | 37.7 | 37.2 | 33.0 | 27.5 | 20.3 | 9.9 |
| **Mean minimum temperature (℃)** | -17.9 | -13.6 | -5.0 | 2.6 | 8.7 | 14.6 | 19.2 | 18.5 | 11.2 | 2.8 | -4.6 | -13.5 |
| **Extreme minimum temperature (℃)** | -29.7 | -28.2 | -26.0 | -7.7 | -2.2 | 6.9 | 10.9 | 8.7 | 0.2 | -7.8 | -20.0 | -28.2 |
| **Mean precipitation (mm)** | 11.1 | 10.9 | 19.9 | 48.5 | 75.0 | 121.7 | 219.0 | 202.7 | 81.9 | 45.2 | 34.7 | 17.4 |
| **Precipitation days (d)** | 7.2 | 6.0 | 7.0 | 9.8 | 12.7 | 14.9 | 17.4 | 14.6 | 10.1 | 8.4 | 9.3 | 8.9 |
| [Mean wind speed](javascript:;) (m/s) | 0.9 | 1.3 | 1.9 | 2.3 | 2.1 | 1.7 | 1.3 | 1.2 | 1.2 | 1.3 | 1.2 | 0.9 |

**S1 Table. Basic climatic information of Ji 'an, Jilin Province, China (According to the data from 1971-2000)**
